# Fatigue and associated factors in men with rheumatoid arthritis: a case-control study using the FACIT-F scale

**DOI:** 10.3389/fmed.2025.1555089

**Published:** 2025-04-28

**Authors:** Joan M. Nolla, Lidia Valencia-Muntalà, Laura Berbel-Arcobé, Diego Benavent, Paola Vidal-Montal, Martí Aguilar-Coll, Montserrat Roig-Kim, Javier Narváez, Carmen Gómez-Vaquero

**Affiliations:** Department of Rheumatology, IDIBELL-Hospital Universitari de Bellvitge, University of Barcelona, Barcelona, Spain

**Keywords:** fatigue, men, rheumatoid arthritis, FACIT-F scale, comorbidity, case-control study

## Abstract

**Introduction:**

Fatigue is a debilitating condition commonly reported in rheumatoid arthritis (RA), yet its prevalence and associated factors in men remain underexplored. This study investigates the prevalence and severity of fatigue in Spanish men over 50 years with RA.

**Methods:**

A case-control study was conducted at a university hospital, comprising 84 RA patients (mean age: 71.9 ± 8.5 years) and 102 age-matched controls. Fatigue was assessed using the FACIT-F scale, together with evaluations of inflammatory markers (CRP, ESR), disease activity (DAS28, RAPID3), disability (HAQ), and health-related quality of life (SF-12). Group differences were examined, and correlations were analyzed to investigate associations between fatigue and RA-related parameters. Stepwise regression analysis was performed to identify independent predictors of fatigue.

**Results:**

Rheumatoid arthritis patients exhibited greater fatigue compared to controls, with a median FACIT-F score of 41.5 (38; 46.8) versus 46.25 (38; 49) (*p* < 0.05). Fatigue was prevalent among RA patients and showed negative correlations with inflammatory markers (ESR: r = −0.285, *p* < 0.01; CRP: r = −0.232, *p* < 0.01) and disease activity indices (DAS28: r = −0.330, *p* < 0.01; RAPID3: r = −0.475, *p* < 0.01). Positive correlations were observed with the SF-12 physical (r = 0.465, *p* < 0.01) and mental health components (r = 0.438, *p* < 0.01). RAPID3, SF-12, and ESR were the primary predictors of fatigue, collectively explaining up to 42.1% of its variance.

**Conclusion:**

Fatigue is a significant comorbidity in men with RA, closely linked to inflammation, disease activity and reduced quality of life.

## Introduction

Rheumatoid arthritis (RA), the most prevalent systemic autoimmune disease worldwide, manifests as a complex condition with persistent and progressive joint and extra-articular symptoms, significantly increasing the risk of disability and mortality (1).

In Spain, the prevalence of RA is estimated at approximately 0.5% of the adult population, with higher rates observed in women and urban areas (2). Patients are predominantly managed in specialized hospital-based rheumatology services, where routine follow-up and therapeutic decision-making are carried out in accordance with national and international clinical guidelines.

The broad spectrum of RA manifestations amplifies its overall impact, emphasizing the critical need for continuous and effective disease management strategies that also address patient comorbidities (3). These associated conditions play a crucial role in determining the prognosis of RA.

Fatigue is characterized by an overwhelming sense of exhaustion that adversely affects the patient’s quality of life and severely impairs their ability to perform daily activities (4). The nature of this fatigue is complex, involving a combination of physiological, psychological, and social factors (5).

Fatigue is a frequently reported and disabling symptom in RA, with a broad impact on daily functioning, emotional well-being, and social participation. It is consistently prioritized by patients within their top outcome priorities, often as high or higher than pain (6).

Despite its significant impact, fatigue is often underestimated in rheumatological practice. In RA, we recently conducted the first case-control study addressing this issue, demonstrating that fatigue remains highly prevalent and severe among women over 50 years in a clinical setting (7). The study employed the Functional Assessment of Chronic Illness Therapy-Fatigue (FACIT-F) questionnaire, a widely recognized tool for assessing fatigue in chronic diseases (8).

There has been limited analysis of fatigue in men with RA (9–11). Moreover, there are no case-control studies that allow for comparisons with the general population.

This study aimed to assess the prevalence and the severity of fatigue among a cohort of Spanish men with RA using the FACIT-F scale and to compare these findings with a control group. Additionally, we explored the relationship between FACIT-F scores and critical RA-related variables to identify key determinants of fatigue. Ultimately, our objective was to evaluate the clinical relevance of this comorbidity in this population and to assess the necessity of incorporating systematic analysis of this condition into routine clinical practice.

## Methods

The study protocol followed the same methodology as our previous research conducted on women (7).

### Study population

This observational case-control study focused on men over 50 years old with RA, comparing them to age-matched controls. All patients in the RA group had a confirmed diagnosis based on the 2010 ACR/EULAR classification criteria and were under regular follow-up in the rheumatology outpatient clinic at our hospital. Controls were selected through three primary sources: accompanying individuals of patients attending the rheumatology service, individuals with non-inflammatory musculoskeletal disorders (primarily soft tissue conditions), and those visiting the hospital for reasons unrelated to musculoskeletal diseases.

To ensure comparability, participants in both groups were screened to exclude any conditions known to cause fatigue, including cancer, heart or respiratory failure, chronic liver or kidney diseases, and central sensitivity syndromes. All participants provided written informed consent, and the study received approval from the local ethics committee (reference: PR057/20).

### Study variables

#### Sociodemographic and anthropometric data

• Age
• Body Mass Index (BMI): Calculated as the ratio of body weight to squared height, expressed in kg/m^2^. BMI categories were defined as follows:
  ◦ < 18.5 kg/m^2^: underweight
  ◦ 18.5–24.9 kg/m^2^: normal range
  ◦ 25–29.9 kg/m^2^: overweight
  ◦ ≥ 30 kg/m^2^: obese
• Tobacco Use: Participants were classified into three groups based on smoking status:
  ◦ Never smokers
  ◦ Current smokers
  ◦ Former smokers
• Physical Activity: Categorized into four levels based on frequency and intensity:
  ◦ None
  ◦ Sporadic
  ◦ Regular with low intensity
  ◦ Regular with high intensity

#### Fatigue assessment

The FACIT-F scale (12) was employed to measure fatigue levels. This scale includes items rated on a scale from 0 to 4, yielding a total possible score that ranges from 0 to 52, where lower scores signify greater levels of fatigue. While there is no universally accepted cutoff for the presence of fatigue, for the purposes of our study, we pre-established a score below 40 to denote “fatigue” which is in line with the several studies available in the literature (9, 10).

#### Evaluation of health-related quality-of-life

We employed the SF-12 questionnaire (13). The SF-12, or Short Form Health Survey, is a 12-item questionnaire designed to measure health-related quality of life. It assesses functional health and well-being from the patient’s perspective. The SF-12 includes two composite scores representing physical and mental health. It is a condensed version of the SF-36 survey, aimed at reducing the burden on respondents while preserving essential health status information. For each participant, two summary scores were calculated: one for physical health and another for mental health. The scores range from 0 to 100, where a higher value indicates a better health-related quality of life.

#### RA assessment

• Evaluation of RA history: (a) disease duration; (b) positivity of rheumatoid factor (RF), along with their titers; (c) positivity for anti-citrullinated peptide antibodies (ACPA), along with their titers; (d) current treatment (glucocorticoids, conventional disease-modifying antirheumatic drugs, biological disease-modifying antirheumatic drugs, Jak inhibitors).
• Analytical evaluation. We considered the following parameters: a) albumin levels; b) erythrocyte sedimentation rate (ESR); c) C-reactive protein (CRP); and d) hemoglobin levels. The values corresponding to the last analytical study carried out were considered. These parameters were not measured in the control group, since all controls presented with non-inflammatory musculoskeletal complaints and had no clinical indication for laboratory testing.
• Evaluation of RA activity using metrological indices. We utilized two indices for this purpose: a) the Disease Activity Score 28 (DAS28) and b) the Routine Assessment of Patient Index Data 3 (RAPID3).
• DAS28 (14) is a composite index of disease activity comprising tender and swollen joint counts in 28 joints, the Patient’s Global Assessment of Disease Activity and the ESR. The higher the score, the higher the activity level. A value < 2.6 suggests disease remission, a value between ≥ 2.6 and ≤ 3.2 suggests low disease activity, a value > 3.2 ≤ 5.1 suggests moderate disease activity and, finally, a value > 5.1 suggests high disease activity.
• RAPID3 (15) is a validated index for measuring disease activity in patients with RA that includes three measures self-reported by the patient: pain, physical function, and global assessment of the disease. The higher the score, the higher the activity level. A value ≤ 3 suggests disease remission, a value between 3.01 and 6 suggests low disease activity, a value between 6.01 and 12 suggests moderate disease activity and a value > 12 suggests high disease activity.
• Evaluation of disability. We used the Health Assessment Questionnaire (HAQ) (16). This questionnaire assesses physical functioning as difficulty performing daily living activities; the score ranges from 0 to 3. The higher the score, the higher the disability level.

### Statistical analysis

To determine the sample size necessary for our case-control study, we utilized the following parameters: a prevalence of fatigue estimated at 50% among cases and 30% among controls, a significance level (alpha) set at 0.05, and a power (1 - beta) of 0.8, reflecting an 80% power to detect a significant difference. The ratio of cases to controls was established at 1:1. The calculations indicated that a sample size of 93 cases and 93 controls, totaling 186 participants, was required to detect a significant difference in fatigue prevalence between the case group (men with RA) and the control group, at the specified significance and power levels.

Data are presented as the mean plus or minus the standard deviation/median and interquartile range for continuous variables and as a number and percentage for categorical variables. Prevalence rates are given as percentages. Normality was assessed using the Kolmogorov-Smirnov test for the FACIT-F scores, the primary outcome variable. Based on this assessment, the median was employed as the primary measure of central tendency for FACIT-F due to its non-normal distribution, ensuring a robust representation and minimizing the influence of outliers. Differences among parametric variables were assessed using ANOVA; for non-parametric variables, we used the U-Mann-Whitney or Kruskal-Wallis tests, when indicated. Differences among categorical variables were evaluated by the chi-squared test.

To assess the relationship between the variables of interest in this study, a correlation analysis using Pearson’s correlation coefficient was conducted. This analysis allowed us to examine linear associations between pairs of variables and determine the strength and direction of these relationships. A multivariate study by multiple regression including all the variables that correlated with FACIT-F plus age, BMI, and RA disease duration was used to identify independent factors influencing fatigue.

## Results

Table 1 summarizes the characteristics of the 84 RA patients included in the study. The cohort had an average age of 71.9 ± 8.5 years and a mean BMI of 27.5 ± 3.5 kg/m^2^. The mean disease duration was 12.5 ± 9.6 years. DAS28 scores indicated that 58.3% of patients were in remission, while 17.9% had moderate disease activity. RAPID3 scores showed 44% in remission and 32.1% with moderate disease activity. The HAQ scores reflected low disability, and 86.9% of patients were receiving DMARD therapy. Mean SF-12 mental health and physical health scores were 51.5 ± 10 and 42.5 ± 0.6, respectively.

**TABLE 1:** Characteristics of the patients with rheumatoid arthritis (RA) (*n*: 84) included in the study.

Variables	Results
Age (years)	71.9 ± 8.5
BMI (kg/m^2^)	27.5 ± 3.5
Underweight (*n*, %)	1 (1.2%)
Normal range (*n*, %)	18 (21.4%)
Overweight (*n*, %)	49 (58.3%)
Obese (*n*, %)	16 (19.1%)
Smoking	
Never (*n*, %)	72 (85.7%)
Former (*n*, %)	1 (1.2%)
Current (*n*, %)	11 (13.1%)
Physical activity	
None (*n*, %)	31 (37%)
Sporadic (*n*, %)	16 (19%)
Regular with low intensity (*n*, %)	33 (39.2%)
Regular with high intensity (*n*, %)	4 (4.8%)
Albumin (g/L)	42.9 ± 4.7
Disease duration (years)	12.5 ± 9.6
RF+ (*n*: 83) (*n*, %)	50 (60.2%)
RF titer (only RF+) (UI/L)	175 ± 224
ACPA+ (*n*:83) (*n*, %)	52 (62.6%)
ACPA titer (only ACPA+) (U/L)	571 ± 1040
ESR (mm/h)	24.3 ± 26.2
CRP (mg/dL)	10.1 ± 18.3
Hemoglobin (g/dL)	14.2 ± 1.5
DAS28	2.5 ± 1.2
Remission (*n*, %)	49 (58.3%)
LDA (*n*, %)	16 (19%)
MDA (*n*, %)	15 (17.9%)
HDA (*n*, %)	4 (4.8%)
RAPID3	5.8 ± 5.5
Remission (*n*, %)	37 (44%)
LDA (*n*, %)	11 (13.1%)
MDA (*n*, %)	27 (32.1%)
HDA (*n*, %)	9 (10.8%)
HAQ	0.09 ± 0.19
Current medication	
Glucocorticoids (*n*, %)	46 (54.7%)
cDMARDs (*n*, %)	73 (86.9%)
bDMARDs (*n*, %)	20 (23.8%)
Jak inhibitors (*n*, %)	2 (2.4%)
FACIT-F (median) [IQR]	41.5 [38; 46.8]
FACIT-F ≤ 40 (*n*, %)	27 (32.1%)
SF-12	
Mental health	51.5 ± 10.0
Physical health	42.5 ± 9.6

BMI, body mass index; RF, rheumatoid factor; ACPA, anti-citrullinated peptides antibodies; ESR, erythrocyte sedimentation rate; CRP, C-reactive protein; DAS28, Disease Activity Score 28; LDA, low disease activity; MDA, moderate disease activity; HDA: high disease activity; RAPID3, Routine Assessment of Patient Index Data 3; HAQ, Health Assessment Questionnaire; cDMARDs, conventional disease-modifying antirheumatic drugs; bDMARDs; biological disease-modifying antirheumatic drugs; FACIT-F: Functional Assessment of Chronic Illness Therapy-Fatigue; IQR, interquartile range; SF-12: Short Form Health Survey-12.

Table 2 compares RA patients and controls. The median FACIT-F score in RA patients was 41.5 (38; 46.8), significantly lower than that of the control group, 46.25 (38; 49) (*p* < 0.05). However, no significant differences were observed in the proportion of individuals with FACIT-F scores ≤ 40 between the groups. RA patients exhibited significantly lower SF-12 physical health scores compared to controls (*p* < 0.01).

**TABLE 2:** Comparison of patients with rheumatoid arthritis (RA) and controls.

	Patients (*n*: 84)	Controls (*n*: 102)	*P*
Age (years)	71.9 ± 8.6	71.1 ± 9.2	ns
BMI (kg/m^2^)	27.5 ± 3.5	27.4 ± 4.4	ns
Underweight (*n*, %)	1 (1.2%)	0	–
Normal range (*n*, %)	18 (21.4%)	33 (32.3%)	–
Overweight (*n*, %)	49 (58.3%)	46 (45.1.%)	–
Obese (*n*, %)	16 (19.1%)	23 (22.6%)	ns
Hemoglobin (g/dL)	14.2 ± 1.4	14.5 ± 1.6	ns
FACIT-F (median) [IQR]	41.5 [38; 46.8]	46.25 [38;49]	< 0.05
FACIT-F ≤ 40 (*n*, %)	27 (32.1%)	30 (29.4%)	ns
SF-12			
Mental health	51.5 ± 10.0	50.8 ± 9.9	ns
Physical health	42.5 ± 9.6	46.7 ± 10.6	< 0.01

BMI, body mass index; FACIT-F: Functional Assessment of Chronic Illness Therapy-Fatigue; IQR, interquartile range; SF-12: Short Form Health Survey-12.

Table 3 highlights the association between fatigue and disease burden in RA patients. Patients with fatigue exhibited significantly higher disease activity, greater disability, and poorer quality of life compared to those without fatigue.

**TABLE 3:** Differences between patients without and with fatigue.

	Without fatigue (*n*: 57)	With fatigue (*n*: 27)	*P*
Age (years)	71.4 ± 8.6	73.0 ± 8.6	ns
BMI (kg/m^2^)	27.3 ± 3.3	28.0 ± 3.8	ns
Underweight (*n*, %)	1 (1.8%)	0	–
Normal range (*n*, %)	12 (21%)	6 (22.2%)	–
Overweight (*n*, %)	34 (59.7%)	15 (55.6%)	–
Obese (*n*, %)	10 (17.5%)	6 (22.2%)	ns
Smoking			
Never (*n*, %)	48 (84.2%)	24 (88.8)	–
Ever (*n*, %)	1 (1.8%)	0	–
Current (*n*, %)	8 (14%)	3 (11.2)	ns
Physical activity			
None (*n*, %)	20 (35.1%)	11 (40.7%)	–
Sporadic (*n*, %)	11 (19.3%)	5 (18.6%)	–
Regular with low intensity (*n*, %)	23 (40.4%)	10 (37%)	–
Regular with high intensity (*n*, %)	3 (5.2%)	1 (3.7)	ns
Albumin (g/L)	43.5 ± 4.3	41.6 ± 5.3	ns
Disease duration (years)	12.4 ± 8.7	12.8 ± 11.6	ns
RF+ (*n*: 83) (*n*, %)	26 (45.6%)	24 (88.8%)	< 0.001
RF titer (only RF+) (UI/L)	145 ± 174	211 ± 271	ns
ACPA+ (*n*: 83) (*n*, %)	30 (52.6%)	22 (81.5%)	< 0.05
ACPA titer (only ACPA+) (U/L)	562 ± 911	583 ± 1220	ns
ESR (mm/h)	18.1 ± 18.7	37.6 ± 34.1	< 0.01
CRP (mg/dL)	7.3 ± 12.6	15.9 ± 25.8	< 0.05
Hemoglobin (g/dL)	14.4 ± 1.3	13.7 ± 1.7	< 0.05
DAS28	2.2 ± 1.0	3.1 ± 1.4	< 0.01
Remission (*n*, %)	40 (70.2%)	9 (33.3%)	–
LDA (*n*, %)	10 (17.5%)	6 (22.2%)	–
MDA (*n*, %)	6 (10.5%)	9 (33.3%)	–
HDA (*n*, %)	1 (1.8)	3 (11.2%)	< 0.01
RAPID3	4.7 ± 4.9	8.2 ± 5.9	< 0.01
Remission (*n*, %)	30 (52.7%)	7 (25.9%)	–
LDA (*n*, %)	8 (14%)	3 (11.2%)	–
MDA (*n*, %)	16 (28.1%)	11 (40.7%)	–
HDA (*n*, %)	3 (5.2%)	6 (22.2%)	< 0.05
HAQ	0.07 ± 0.18	1.1 ± 0.19	< 0.01
Current medication			
Glucocorticoids (*n*, %)	31 (54.4%)	15 (55.6%)	ns
cDMARDs (*n*, %)	48 (84.2%)	25 (92.5%)	ns
bDMARDs (*n*, %)	14 (24.6%)	6 (22.2%)	ns
Jak inhibitors (*n*, %)	1 (1.8%)	1 (3.7%)	ns
SF-12			
Mental health	54.2 ± 8.5	46.0 ± 11.0	< 0.001
Physical health	44.5 ± 9.3	38.4 ± 9.1	< 0.01

BMI, body mass index; RF, rheumatoid factor; ACPA, anti-citrullinated peptides antibodies; ESR, erythrocyte sedimentation rate; CRP, C-reactive protein; DAS28, Disease Activity Score 28; LDA, low disease activity; MDA, moderate disease activity; HDA: high disease activity. RAPID3, Routine Assessment of Patient Index Data 3; HAQ, Health Assessment Questionnaire; cDMARDs, conventional disease-modifying antirheumatic drugs; bDMARDs; biological disease-modifying antirheumatic drugs; SF-12: Short Form Health Survey-12.

In RA patients, FACIT-F scores showed significant correlations with clinical and quality of life parameters. Negative correlations were observed with ESR (r = −0.285, *p* < 0.01), CRP (r = −0.232, *p* < 0.01), DAS28 (r = −0.330, *p* < 0.01), and RAPID3 (r = −0.475, *p* < 0.01). Positive correlations were found with SF-12 mental health (r = 0.438, *p* < 0.01) and SF-12 physical health (r = 0.465, *p* < 0.01).

A stepwise multiple regression analysis was performed to identify factors influencing fatigue, as measured by the FACIT-F scale. Variables included those correlated with FACIT-F, along with age, BMI, and RA disease duration. Five models were generated during the analysis. Model 1 incorporated only RAPID3, demonstrating a significant independent negative association with fatigue (B = −0.499, *p* < 0.001). The final model, with an R^2^ of 0.421, revealed that fatigue is significantly associated with inflammatory markers (ESR) and mental and physical health (SF-12), collectively explaining 42.1% of the variance in fatigue. The coefficients and significance levels for each variable in the models are detailed in Table 4.

**TABLE 4:** Multivariate analysis including all the variables that correlated with Functional Assessment of Chronic Illness Therapy-Fatigue (FACIT-F) plus age, body mass index (BMI) and rheumatoid arthritis (RA) disease duration.

	Constant	Coefficient	*P*	*R* ^2^
**Model 1**				
RAPID3	44.596	−0.499	< 0.001	0.239
**Model 2**				
RAPID3	33.020	−0.400	< 0.001	0.330
SF12 MH		0.329	< 0.01	
**Model 3**				
RAPID3	20.478	−0.188	ns	0.402
SF12 MH		0.360	< 0.001	
SF12 PH		0.346	< 0.01	
**Model 4**				
SF12 MH	14.610	0.406	< 0.001	0.389
SF12 PH		0.452	< 0.001	
**Model 5**				
SF12 MH	17.054	0.375	< 0.001	0.421
SF12 PH		0.445	< 0.001	
ESR		−0.200	< 0.05	

RAPID3, Routine Assessment of Patient Index Data 3; SF-12, Short Form Health Survey-12; MH, mental health; PH, physical health; ESR. Erythrocyte Sedimentation Rate.

## Discussion

This study highlights the prevalence, severity, and multifaceted nature of fatigue in older men with rheumatoid arthritis, a population that has received limited attention in rheumatological research. The findings emphasize the significance of fatigue as a critical comorbidity, strongly associated with inflammation, disease activity, and quality of life. These results support the systematic evaluation of fatigue in clinical practice, especially in male patients with active disease, and highlight the importance of incorporating fatigue management into comprehensive treatment strategies.

We selected the FACIT-F scale to evaluate fatigue due to its extensive validation in chronic conditions, including rheumatoid arthritis, and its strong psychometric properties (8). It has demonstrated excellent internal consistency, sensitivity to change, and ease of use across languages and clinical contexts. Although not explicitly multidimensional, the FACIT-F scale captures both physical and emotional domains of fatigue and includes items that indirectly reflect its impact on daily function and quality of life, which are closely related to social participation. It has been increasingly adopted as a patient-reported outcome in recent RA clinical trials (17–19).

In our study, differences in median FACIT-F scores between cases and controls underscore a disparity in fatigue intensity rather than its prevalence. The absence of significant differences in the prevalence of fatigue (FACIT-F ≤ 40) may reflect a high baseline prevalence of fatigue in the general population, particularly among older adults, although the relationship between age and fatigue remains controversial (20). Having a control group is crucial for contextualizing fatigue levels, enabling the identification of variations in fatigue intensity that might otherwise go unnoticed. These findings suggest that, while fatigue is prevalent in both populations, its severity is a distinguishing factor in men with RA, highlighting the need for targeted management strategies aimed at reducing fatigue intensity rather than merely addressing its presence. As expected, individuals in the control group exhibited higher scores in the physical health component of the SF-12 compared to patients, likely reflecting the impact of the disease.

Our data indicate that increased inflammation, disease activity, and perceived disease severity are strongly associated with greater fatigue. Pro-inflammatory cytokines such as IL-6 and TNF-α, which are elevated in active RA, are believed to contribute to central mechanisms of fatigue by modulating neuroimmune pathways. CRP and ESR, while non-specific, reflect systemic inflammation and have been associated with greater fatigue severity in RA, potentially serving as indirect indicators of inflammation-driven fatigue (21). Furthermore, lower fatigue levels were linked to better quality of life.

The multivariate analysis highlights RAPID3 as a pivotal predictor of fatigue in RA patients. The significant negative association between RAPID3 scores and fatigue underscores that higher levels of patient-reported disability and pain are strongly linked to increased fatigue. The consistent significance of RAPID3 across multiple models demonstrates its robustness as a predictor, explaining a substantial proportion of the variance in fatigue.

The decision to focus specifically on male patients with RA was guided by their underrepresentation in fatigue studies and by emerging evidence of sex-related differences in disease activity and patient-reported outcomes (22). Applying a gender-specific perspective may enhance our understanding of variability in fatigue experience and support more personalized management strategies.

Contextualizing the findings with those from our previous study (7) on women with RA (*n* = 191; mean age: 67.5 ± 8.8) reveals notable similarities and differences. Both studies highlight fatigue as a significant comorbidity; however, men generally reported higher FACIT-F scores, indicating slightly lower fatigue intensity compared to women [41.5 (38; 46.8) vs. 38 (30; 43)].

Despite these differences in intensity, the determinants of fatigue showed consistencies across genders, with disease activity, inflammation, and quality of life consistently emerging as key predictors. However, the strength of these associations varied: in women, mental health (as assessed by SF-12) demonstrated a stronger correlation with fatigue, whereas in men, physical health scores appeared to play a more prominent role.

Our findings highlight the importance of considering gender differences in the experience of fatigue among patients with RA in clinical contexts. This observation aligns with those reported by other authors in studies conducted on specific populations, such as patients categorized by early RA or by methotrexate usage.

Thyberg et al. (23), in a longitudinal study in Sweden with 276 patients with early RA (191 women, 85 men; mean age: 54 ± 15 years in women and 58 ± 14 years in men) and a disease duration of less than 1 year, examined gender differences in fatigue [measured using the *Visual Analog Scale* (VAS)] and its associations with disease activity, pain, sleep disturbances, mental health, and activity limitations. Women reported slightly higher levels of fatigue compared to men, along with greater activity limitations and lower mental health scores. Similarly, Bay et al. (24), in a Danish study of 286 patients with RA (217 women, 69 men; mean age: 56.6 ± 10.0 years; mean disease duration: 12.1 years), of whom 67.8% were methotrexate (MTX) users, focused on gender differences in fatigue [assessed using the VAS and the *Bristol Rheumatoid Arthritis Fatigue Numerical Rating Scales* (BRAF-NRS)], depression, physical function, loneliness, and sexual dysfunction. Women exhibited higher levels of fatigue, depression, and disability compared to men, both among MTX users and non-users.

This research has several limitations. The study included 84 cases and 102 controls, falling slightly short of the initially estimated 93 cases. Although this modest reduction may have limited the ability to detect some associations, the sample size remained close to the calculated threshold for statistical power. The inclusion of additional controls helped to maintain the robustness of the comparisons and mitigate this limitation. The single-center design raises concerns about the generalizability of our findings, but we believe our cohort reflects the characteristics of patients with long-standing RA typically managed in university hospital settings.

Although we applied strict exclusion criteria to rule out conditions potentially associated with fatigue in the control group, the recruitment of participants from a hospital setting may still have introduced a degree of selection bias. Nonetheless, this approach allowed for clinical screening and accurate exclusion of confounding pathologies, which would have been more difficult to ensure in a general population sample.

Depression, a known factor associated with higher fatigue scores in RA (25) was not systematically evaluated in this study. The absence of formal assessment may have limited our ability to account for its potential contribution to the fatigue reported by some participants. Furthermore, although conditions associated with fatigue were excluded, we did not provide a detailed description of comorbidities in either group, which may have influenced the findings.

As with any cross-sectional study, causal relationships between RA characteristics and fatigue cannot be established. Additionally, the absence of a direct comparative design limits conclusions regarding gender differences in fatigue. Nevertheless, our data reflect real-world clinical practice, capturing the heterogeneity of unselected patient populations and offering valuable insights for routine care.

Our findings emphasize the critical role of fatigue in the overall disease burden within this demographic. The observed associations with disease activity, as assessed by DAS28 and RAPID3, reinforce its importance as a therapeutic target. By focusing on a population often underrepresented in rheumatological research, our study provides valuable insights into gender-specific aspects of fatigue, contributing to a more nuanced understanding of its impact in RA. The use of validated instruments, such as the FACIT-F scale, DAS28, RAPID3 and SF-12, ensures the robustness and reliability of the data.

Furthermore, the inclusion of a control group enhances the contextualization of fatigue levels, highlighting the disparities in fatigue intensity between RA patients and the general population.

Our findings support integrating systematic assessment and targeted management of fatigue into routine clinical care. Rather than treating it as a secondary or non-specific complaint, fatigue should be recognized as a clinical target. Elevating fatigue to this status may promote a more comprehensive approach and ultimately improve patient outcomes in RA.

Future research should prioritize the development of targeted interventions to mitigate the impacts of fatigue in RA. Expanding studies with longitudinal designs will offer deeper insights into the underlying mechanisms of this condition and support the advancement of personalized management strategies tailored to the needs of RA patients.

## Data Availability

The raw data supporting the conclusions of this article will be made available by the authors, without undue reservation.
